# Cognitive and Non-Cognitive Science Gains from SEL Intervention in Arabic-Speaking Students: Comparing Typical and Struggling Readers

**DOI:** 10.3390/jintelligence14060104

**Published:** 2026-06-10

**Authors:** Ahmad Basheer, Ibrahim A. Asadi

**Affiliations:** 1 Department of Science Education, The Arab Academic College for Education in Israel, Haifa, #22 Hachashmal St., Haifa 33145, Israel; 2 Department of Special Education and Learning, The Arab Academic College for Education in Israel, Haifa, #22 Hachashmal St., Haifa 33145, Israel

**Keywords:** academic achievement, Arabic-speaking students, intervention study, motivation, reading difficulties, science education, social and emotional learning

## Abstract

This experimental study investigated the impact of embedding social and emotional learning (SEL) in science instruction on the academic and social–emotional outcomes of Arabic-speaking sixth graders, including those with reading difficulties (RD). Children from two schools in northern Israel (*n* = 101) were randomly assigned to either an intervention group, which received SEL-enriched science lessons featuring collaborative, reflective activities over 30 sessions, or a control group receiving traditional instruction. Pre- and post-tests assessed SEL competencies, motivation towards science, and academic achievements in science and mathematics. Results showed significantly greater gains in SEL skills, and in science motivation and science achievement in the intervention group compared to controls, whereas mathematics outcomes remained unchanged. Typically developing students and those with RD benefited similarly. Integration of SEL into science curricula thus enhances cognitive and social–emotional learning dimensions, particularly in linguistically and socio-economically marginalised populations. Implications for inclusive pedagogy and future research directions are discussed.

## 1. Introduction

The growing recognition of social and emotional learning (SEL) as a critical foundation for students’ academic and lifelong success has prompted extensive empirical inquiry worldwide (Durlak et al., 2022; Prince, 2024). SEL, encompassing competencies such as self-awareness, self-regulation, social awareness, relationship skills, and responsible decision-making, has been linked with diverse positive outcomes, including improved classroom behaviour, enhanced emotional wellbeing, and higher academic achievement (Collaborative for Academic, Social, and Emotional Learning, 2023; Cipriano et al., 2023). The importance of these competencies extends beyond the social domain, influencing learning engagement, resilience, and academic persistence across subjects.

Contemporary research demonstrates that academic achievement arises from a dynamic interplay between cognitive abilities (e.g., memory, executive function, linguistic skills) and social–emotional factors (Duckworth & Seligman, 2005; Ryan & Deci, 2009). Skills in mathematics and science, in particular, frequently pose unique challenges for elementary learners (Basheer et al., 2025a). These subjects often demand abstract reasoning, structured problem-solving, the comprehension of lexically dense texts, and the ability to manipulate symbolic representations (Fang, 2006; Organisation for Economic Co-Operation and Development, 2019). The complexity and unfamiliarity of scientific texts, coupled with technical vocabulary and abstract concepts, can further hinder comprehension and motivation, particularly among learners with reading difficulties (RD) (Perfetti & Stafura, 2014).

Recent pedagogical models emphasise the importance of supporting not only cognitive skill development, but also the social–emotional context in which learning takes place. Interventions targeting SEL have boosted academic outcomes, classroom climate, and student motivation (Fund & Madjar, 2018; Wigfield et al., 2016). Such effects are especially pronounced in linguistically and socio-economically marginalised populations, such as Arabic-speaking children, who often encounter the dual barriers of complex orthographic systems and limited experiential resources. Beyond these pedagogical and linguistic considerations, neuroscientific work suggests that language and social cognition are closely interconnected capacities, supported by partially overlapping neural systems that evolved under pressures of social complexity (Oesch, 2024). This perspective offers a biological rationale for integrating social–emotional processes into language-intensive academic learning and is especially pertinent for children contending with complex linguistic environments.

Despite the documented benefits of SEL-enriched instruction, research gaps remain regarding the unique contribution of SEL interventions to facilitating science and mathematics learning among Arabic-speaking children with and without RD (Basheer et al., 2025a; Hofstein & Mamlok-Naaman, 2007). Three interrelated gaps motivate the present study. First, the meta-analysis of universal SEL programs by Cipriano et al. (2023) draws largely on interventions in Western, English-speaking contexts, so the efficacy of SEL-integrated instruction in Arabic-speaking educational settings is still largely untested. Second, although Basheer et al. (2025a) examined how laboratory-based experiences shape science–text comprehension and motivation among Arabic-speaking children, their design neither embedded SEL components in the science curriculum nor distinguished typically developing children from those with RD. Third, Asadi (2025) showed that teachers’ reports of students’ SEL competencies predict academic performance in Arabic-speaking children; the evidence, however, is cross-sectional and correlational, and cannot establish whether a targeted SEL intervention itself produces gains in science and mathematics learning. These three gaps motivated us to examine whether a structured, curriculum-integrated SEL intervention delivered within science instruction will produce cognitive and social–emotional gains for Arabic-speaking sixth graders, with particular attention to typical readers and those with RD, a group for whom tailored support may be especially impactful.

The literature review synthesises current evidence regarding: (a) the cognitive and social–emotional foundations of academic learning, especially in mathematics and science; (b) the specific challenges presented by these domains and by the Arabic language; (c) the mechanisms through which SEL influences academic and social outcomes; and (d) the efficacy of intervention programs, especially in multi-cultural, multi-lingual settings. It concludes by articulating the significance of the current intervention and presenting the specific research questions addressed.

### 1.1. Literature Review

Academic achievement is fundamentally rooted in a constellation of domain-general and domain-specific cognitive skills, such as working memory, executive functioning, and linguistic proficiency (Baddeley, 2003). Reading, mathematics, and science each rely on both shared and unique learning processes, usually carried out and assessed by comprehending written material, i.e., reading comprehension. The Simple View of Reading posits that reading comprehension requires both word recognition and oral language comprehension, a model that is supported across languages, including Arabic (Asadi & Kasperski, 2024; Gough & Tunmer, 1986). In mathematics and science, conceptual understanding requires not only procedural fluency, but also abstract reasoning and the integration of multi-sensory information (Brigham et al., 2011; Holyoak & Spellman, 1993).

Understanding the contribution of SEL to academic learning requires first considering the cognitive, linguistic, and motivational demands characteristic of science and mathematics instruction.

#### 1.1.1. Cognitive, Linguistic, and Motivational Foundations of Learning in Science and Mathematics

Science and mathematics are often perceived as especially challenging due to their reliance on unfamiliar concepts, technical terminology, and specific modes of thinking (Fang, 2006). Scientific texts, for example, are characteristically lexically dense and abstract, frequently utilising nominalised forms and complex structures. This complexity presents barriers not only for students with RD but for all learners, as it necessitates the integration of new information with prior knowledge, often with minimal scaffolding from the text (Carlisle, 1999; Bin Sawad et al., 2022). For Arabic-speaking children, additional obstacles stem from diglossia, orthographic complexity, and the infrequent alignment of spoken and written forms (Asli-Badarneh, 2025; Asadi & Asli-Badarneh, 2023; Saiegh-Haddad & Joshi, 2014).

While cognitive skills, including decoding, working memory, and linguistic knowledge are central to learning, there is a growing body of research on the significant influence of social–emotional factors, such as motivation, classroom climate, and social–emotional competencies, on educational outcomes (Duckworth & Seligman, 2005; Wang & Eccles, 2013). Motivation, particularly students’ beliefs about their competence and the value of learning, predicts engagement and persistence in challenging subjects (Ryan & Deci, 2009; Wigfield et al., 2016). Furthermore, a positive classroom climate is linked to greater emotional wellbeing, resilience, and sustained academic effort (Wang & Degol, 2016; Wang & Eccles, 2013).

#### 1.1.2. Social and Emotional Learning as a Mechanism for Academic Engagement and Achievement

Research on social–emotional competencies in education connects closely to a wider literature within intelligence research on non-cognitive dimensions of ability. In this tradition, social–emotional skills have been studied as constructs conceptually related to classical intelligence (Zeidner et al., 2002) and as predictors of academic outcomes that operate alongside cognitive ability, rather than displacing it. MacCann et al. (2020), in a meta-analysis of emotional intelligence as an ability construct, reported that social–emotional abilities account for variations in academic performance over and above intelligence and personality, with three candidate mechanisms: regulation of academic emotions, management of classroom relationships, and partial overlap between social–emotional and curricular content. Corcoran et al. (2018), in a meta-analysis spanning 50 years of school-based SEL research, found positive effects on reading, mathematics, and science achievement. Taken together, these findings point to a systematic link between social–emotional competencies and learning across academic domains. In the present study, SEL is not treated as a statistical mediator set against a competing cognitive pathway; it is treated as an instructional design principle that shapes the affective and collaborative conditions under which domain-specific learning takes place. The expected effects are most visible in science, where text-based and discussion-intensive work draws heavily on emotional regulation and relational engagement.

Within this broader understanding of learning as both a cognitive and motivational process, SEL refers to programs and processes designed to develop five interrelated sets of cognitive, affective, and behavioural competencies: self-awareness, self-management, social awareness, relationship skills, and responsible decision-making (Collaborative for Academic, Social, and Emotional Learning, 2023). Research indicates that integrating SEL into the curriculum benefits academic achievement, pro-social behaviour, and emotional health (Cipriano et al., 2023; Fund & Madjar, 2018). In a recent cross-sectional study of 241 Arabic-speaking students from elementary and middle schools, teachers’ SEL reports about their students significantly predicted the students’ academic scores in mathematics, science and Arabic (Asadi, 2025). In science education, experiential and collaborative approaches that foster SEL, such as laboratory experiments, group tasks, and reflective discussions, have been shown to enhance motivation, comprehension, and classroom climate (Gericke et al., 2022; Hofstein & Mamlok-Naaman, 2007).

Multiple intervention studies, including some in Arabic-speaking contexts (Cipriano et al., 2023; Basheer et al., 2025a), have demonstrated that SEL programmes yield measurable gains in both social–emotional competencies and academic performance, particularly in reading comprehension, science, and mathematics. Programs that provide multi-sensory and hands-on experiences support cognitive development and offer alternative pathways for students experiencing difficulties with conventional instruction (Ritchey & Goeke, 2006; Stevens et al., 2021). For children with RD, SEL-enriched multi-sensory approaches have proven especially effective in bridging gaps in linguistic and cognitive performance (Gough & Tunmer, 1986).

Studies consistently find that academic progress varies according to students’ cognitive profiles and baseline abilities. Children with RD struggle disproportionately with the dual demands of word-level decoding and higher-order comprehension especially in science-based texts (Basheer et al., 2025a; Saiegh-Haddad, 2018). The Matthew effect describes the compounding of early deficits over time, leading to persistent gaps between typical and struggling learners (Stanovich, 2004). Approaches that individualise support and foster resilience through SEL are essential for closing these achievement gaps (Stevens et al., 2021).

These challenges are particularly salient in Arabic-speaking educational contexts, where language-related factors add an additional layer of complexity to academic learning. Arabic orthography is characterized by visual and morphological complexity, and diglossic variation complicates the correspondence between children’s spoken language and the formal written variety encountered in school texts (Asadi et al., 2017; Asadi & Asli-Badarneh, 2023); scientific and informational texts in Arabic often employ vocabulary, syntactic structures, and discourse conventions that diverge markedly from everyday spoken usage, thereby increasing the cognitive and linguistic load placed on learners. For students with RD, these linguistic characteristics may further exacerbate difficulties in comprehension and engagement, underscoring the importance of instructional frameworks that simultaneously address linguistic, cognitive, and social–emotional dimensions of learning (Saiegh-Haddad, 2018).

#### 1.1.3. The Present Study

Taken together, the evidence reviewed above highlights both the potential of SEL-based instruction and the particular vulnerabilities of Arabic-speaking learners, especially those with RD, when engaging with science and mathematics content. The present study is distinguished by its culturally responsive intervention, structured experimental design, and attention to both cognitive and social–emotional outcomes. By evaluating the effectiveness of SEL integration in science instruction, the study addresses critical gaps in the literature and provides empirically grounded recommendations for educational practice in linguistically diverse settings.

The primary research question guiding this study is as follows: how does the integration of SEL into science instruction influence the academic performance, motivation, and SEL competencies of Arabic-speaking children, and does this influence differ between typically developing children and those with RD?

## 2. Material and Methods

The study consisted of a pre–post quasi-experimental design with an intervention and a control group, conducted with Arabic-speaking sixth-grade children in mainstream elementary schools in Israel. Random allocation was at the class level: in each of two participating schools, two intact classes were randomly assigned, one to the intervention and the other to the control condition. All participants completed pre- and post-intervention measures covering four outcomes: social–emotional competencies, motivation towards science, science achievement, and mathematics achievement. The design therefore combined time (pre-intervention, post-intervention) as a within-subjects factor with group (intervention, control) as a between-subjects factor. A further between-subjects factor, reader profile (typically developing, RD), was based on a median split of the pre-intervention Arabic reading composite and entered the subgroup analyses reported below. The intervention lasted 10 consecutive weeks within the school year and comprised thirty 45 min science lessons, three per week, delivered in both conditions by the regular classroom teacher. Intervention lessons embedded the SEL components described below; control lessons covered the same scientific content without these components. Ethical approval and parental consent were obtained before data collection (see Section 2.1).

### 2.1. Participants

The sample comprised 101 Arabic-speaking children (51 boys) from the sixth grade (*M*_age_ = 12.1 years; *SD* = 0.27). Participants were sampled from two mainstream Arabic elementary schools in separate villages in northern Israel, both characterised by their low socio-economic background. The two schools were classified as of low socio-economic status by the Israeli Ministry of Education. This classification draws on three indicators: parents’ average income, parental occupation level, and the socio-economic ranking of the family’s residential area. Both schools sit within the lowest strata on the national socio-economic index of local authorities. This is in line with wider evidence on Arabic-speaking villages in the northern periphery of Israel, which show systematically lower socio-economic status indicators than the Jewish majority (Agbaria, 2020; Saffuri, 2025). Two classes were sampled from each school, with one class (about 25 children per class) randomly assigned to the intervention group (50 children from the two schools), and the other to the control group (51 children from the two schools). In addition, based on preliminary general Arabic language proficiency scores, the whole sample (intervention and control groups) was divided into two groups: typically developing children (*n* = 51) and those with RD (*n* = 50).

This classification was determined by the median scores on pre-intervention (pre-test) Arabic-language tests, and did not address clinical diagnoses of neurodevelopmental reading disorders (e.g., dyslexia or specific learning disability); no IQ-achievement discrepancy or standardised clinical cut-off was used. Instead, the approach was functional and research-based, in line with the Response-to-Instruction (RTI) framework, within which children scoring below the median of their peer group on curriculum-based reading indicators are labelled “poor readers” for the purpose of studying differential responses to instruction (Fuchs et al., 2004; Vellutino et al., 1996). In this tradition, a median split on pre-intervention reading identifies children who may benefit from, or need, additional instructional support, with no claim of a constitutional or neurodevelopmental origin of the difficulty. The Arabic score used for grouping was taken from the children’s most recent trimester report and averaged three components assessed by classroom teachers under the national curriculum: reading accuracy (oral reading of isolated words and connected text), reading fluency (correct words read per minute), and reading comprehension (comprehension questions on narrative and informational texts). These assessments were part of the ordinary school evaluation schedule and followed the content specifications of the sixth-grade Arabic curriculum of the Israeli Ministry of Education; component scores were recorded on the Israeli 0–100 scale, and the composite Arabic score was the simple average of the three. None of the children in the RD group had a formal diagnosis of a specific learning disability on school record, consistent with the functional, non-clinical nature of the grouping. All of the children in the selected classes participated in this study, excluding those with physical or mental disabilities as indicated in school reports.

The study received approval from the ethics committee of the Arab Academic College for Education in Haifa, Israel (Approval Code: AACE-SE-91224.3; Approval Date: 9 December 2024). In addition, parental consent was obtained for all participating children. Before data collection, parents or legal guardians received a written consent form setting out the aims, procedures, and scope of the research. The form made clear that participation was voluntary, that children could withdraw at any point without consequence, that the data would be used only for scientific and academic purposes, that all information would remain confidential and no identifying details would be disclosed, and that the research activities carried no known risks. Written informed consent was then obtained from the parent or legal guardian of each participating child. The study followed the Declaration of Helsinki and the ethical guidelines of the Arab Academic College for Education in Israel.

### 2.2. Material

The study included measures for SEL and motivation towards science. In addition, children’s academic scores in Arabic language, mathematics, and science were obtained from school administrative records for the most recent trimester.

#### 2.2.1. Social and Emotional Learning (SEL)

SEL competencies were evaluated using an online self-report questionnaire (Google Form) adapted from a previous study (Asadi, 2025; Collaborative for Academic, Social, and Emotional Learning, 2023) that tested the CASEL model. The questionnaire consisted of 40 items designed to measure the 5 sub-scales of the CASEL model: self-awareness, self-regulation or self-management, social awareness, relationship skills, and responsible decision-making (8 items each). Sample items from each sub-scale were “I know how I feel” (self-awareness), “I can control my emotions when I am angry” (self-management), “I try to understand what others feel” (social awareness), “I can work well with other children” (relationship skills), and “I think before I act” (responsible decision-making). The Arabic version had already been developed and validated with Arabic-speaking school-aged children in Israel (Asadi, 2025); this earlier work included a confirmatory factor analysis that supported the five-factor CASEL structure (RMSEA = 0.054, CFI = 0.90), with acceptable sub-scale reliability. The present sample comes from the same linguistic and regional population, so no further translation or re-validation was needed. We report internal-consistency estimates from the present sample alongside the earlier validation. Responses were recorded on a 5-point Likert scale ranging from 1 (totally disagree) to 5 (totally agree). Negatively worded items were reverse-coded, and higher scores indicated greater social–emotional competency. The questionnaire demonstrated high internal consistency (Cronbach’s α = 0.93 at pre-test and 0.96 at post-test).

#### 2.2.2. Motivation

Motivation towards science was assessed using a questionnaire adapted from Glynn et al. (2011) and previously employed by Hugerat et al. (2020). The questionnaire, translated into Arabic and adapted for the target age group, comprised 30 items rated on a 5-point Likert scale (1 = never, 5 = always). The children were required to choose the value that represents their opinion regarding each item. The questionnaire demonstrated good internal consistency (Cronbach’s α = 0.87 at pre-test and 0.90 at post-test). The instrument is part of the Science Motivation Questionnaire family (Glynn et al., 2009, 2011) and taps six motivational dimensions that are central to self-regulated science learning: intrinsic motivation, self-efficacy, self-determination, career motivation, grade motivation, and assessment anxiety. A representative item is “Getting a good grade in science is very important to me.” In the original validation, the questionnaire showed strong internal consistency (Cronbach’s α = 0.93; Glynn et al., 2009), and it has since been used with Arabic-speaking students in Israel in a closely comparable age group, yielding α = 0.88 among eighth-grade learners (Basheer et al., 2025b). The Arabic adaptation used here follows the version previously administered by Hugerat et al. (2020).

In addition, participants’ scores in science, Arabic, and mathematics were examined, as reported in school records. Scores in Arabic encompassed general language skills, including basic reading and higher-order reading comprehension. These scores were used to categorise participants into two groups based on reading proficiency: typically developing children and those with RD. Mathematics scores were included as an additional control variable when assessing the impact of the intervention program on science performance. The intervention program was implemented in science instruction, while no intervention was applied to mathematics education. Mathematics scores were included for two reasons: to test whether intervention gains were confined to the taught subject (domain-specificity), and to see whether an SEL-integrated program in science might carry over to a cognitively adjacent domain. Mathematics instruction in all classes followed the regular sixth-grade Ministry of Education curriculum, without any SEL component. Science and mathematics scores came from the schools’ official records, based on teacher-constructed assessments aligned with the sixth-grade curriculum of the Israeli Ministry of Education, and not on standardised national examinations. All scores were on the Israeli 0–100 scale. The science pre-intervention score was the child’s average on the regular science tests from the trimester before the intervention; the post-intervention score came from a summative science examination at its end. For mathematics, no study-specific tests were developed (since the intervention was confined to science); pre- and post-intervention scores were the children’s average on the regular mathematics tests administered by the mathematics teacher in the same time windows as the science assessments.

### 2.3. Procedure

The study was conducted in three stages. In the first stage (January and February), children’s SEL competencies and motivation were assessed using two separate online questionnaires administered prior to the intervention. These Google Form questionnaires, each taking approximately 10 min to complete, were administered in a computer room at the sampled schools. The two examiners (one per school) were master’s degree students in linguistics and learning disabilities and had received specific and detailed training regarding the administration of the questionnaire. The examiners were blind to the study’s hypotheses and took no part in designing or analysing the intervention. Because the SEL and motivation questionnaires were administered through Google Forms with automatic scoring, the examiners never handled item-level scoring. The children’s academic scores in Arabic, mathematics, and science were drawn directly from the schools’ administrative records (teacher-assigned trimester grades), not produced by the examiners. These safeguards limit the scope of the examiner’s awareness of group membership which might bias the outcome measures.

The second stage (February–May) involved the implementation of the intervention programme, which was designed to integrate social–emotional content into science lessons and followed the formal curriculum for the sixth grade as dictated by the Ministry of Education. One class from each school was randomly assigned to the intervention group, while the other class served as the control group. Control-group children continued with their regular science instruction over the same 10 weeks, with the same number of lessons as the intervention group (three 45 min lessons per week, 30 sessions in total). Control lessons followed the same sixth-grade science curriculum of the Israeli Ministry of Education, covered the same scientific content, and were taught by the regular classroom teacher of each control class. The only systematic difference between the two conditions was the absence, in the control lessons, of the structured SEL components embedded in the intervention, pre-planned open-ended emotional questions, structured cooperative group tasks on social–emotional themes, guided peer discussion on collaborative aspects of the science topic, and an end-of-lesson reflective closing. In other words, the comparison was a business-as-usual active-content control rather than a no-contact one, designed to equate time on task and curricular content across conditions; any post-test differences can therefore be attributed to the SEL-integrated instructional format rather than to differences in science content exposure.

The intervention consisted of 10 study units, developed in collaboration with the school’s educational counsellor, which incorporated social–emotional content into scientific topics. The program was conducted over 30 sessions (three 45 min lessons per week for 10 weeks). Each week focused on one teaching unit, emphasising social and emotional concepts within the scientific context. For example, during a lesson on animal communication, children were prompted to consider the emotional aspects of communication, such as how animals might feel during communication or without any means of communication. They were encouraged to reflect on their own communication strategies and how they might enhance interactions with peers.

The 10 weekly units covered the full sixth-grade science curriculum of the Israeli Ministry of Education (2024): energy types and conversions, light energy, sound energy, electrical energy, energy sources and environmental impact, communication systems in the body (sensory organs and nervous system), healthy lifestyle, ecological systems and biodiversity, interactions between organisms, and human impact on the environment. Every unit followed the same instructional structure for embedding SEL in the scientific content. Each lesson opened with pre-planned open-ended questions that tied the scientific topic to children’s own feelings and perspectives (e.g., “What would you feel if you were in the position of the organism or system being studied?”). It then moved on to structured cooperative tasks in pairs or small groups, where children worked through a question, shared ideas, and presented conclusions. A brief reflective closing rounded off the lesson, with children describing what had engaged or challenged them and how they and their peers had contributed. Table 1 gives five representative examples, one per CASEL competency, from different units of the program.

In addition, small work groups were formed, emphasising teamwork and helping others when needed. Children discussed how they felt after assisting a peer, while those who received help were asked how it made them feel and what impact it had on them. Discussions were also held on how to recognise when someone needs help and what signs indicate that a child might require support. Children also worked on developing structured work strategies individually and collaboratively, experiencing first-hand the effectiveness of planning and monitoring in general, and the value of teamwork in particular. The intervention was delivered in each school by the regular classroom teacher of the intervention class, within the ordinary weekly teaching schedule. Several procedural measures supported the consistency of implementation across the two schools. Before the intervention began, both teachers met with the school’s educational counsellor to select and adapt SEL activities for each of the 10 science units, so that the pedagogical framing was comparable at the two sites. All lessons then followed the same structured format, pre-planned open-ended questions, small-group cooperative tasks, peer discussion, and a brief reflective closing in which children shared their engagement with the day’s activities. Throughout the intervention, the research team stayed in regular contact with the two teachers and the educational counsellor, reviewing and approving the weekly activities and handling implementation questions as they arose. Children’s reflective feedback at the end of each lesson also helped the teachers and the research team track engagement and keep delivery of the 10 units aligned with the protocol.

The third stage, the post-test, was conducted immediately after the intervention program. It tested the performance of both groups in social–emotional competencies and motivation towards science, as in the first stage (pre-test). Like the first stage, children were tested in a computer room by the same examiners to evaluate the impact of the intervention program on cognitive (academic achievements in science) and social–emotional (competencies and motivation towards science) outcomes in both the intervention and control groups.

## 3. Results

The study design involved four intact classes (two per condition) nested within two schools, so students were not fully independent observations. Multi-level modelling (MLM) would normally be the analytic approach of choice for such clustering. In this case, however, the number of level 2 units was well below the minimum typically recommended for stable estimation of between-cluster variance components (at least 10–30 clusters; Maas & Hox, 2005), and MLM was not suitable. We therefore used repeated-measures analysis of variance (ANOVA) as the primary analytic framework, in line with earlier classroom-based intervention studies of comparable scale. The clustered structure of the data remains a limitation, addressed in the Section 4.1.

To ensure the validity of our experimental design, we conducted preliminary analyses to examine potential differences between the intervention and control groups. A series of *t*-tests did not reveal any statistically significant differences between the two groups in gender (*t* = 1.91; *p* > 0.05) or age (*t* = 1.13; *p* > 0.05). In addition, to establish baseline equivalence, a series of *t*-tests were conducted before the implementation of the intervention program. Results indicated no statistically significant differences between groups in SEL competencies (*t* = 1.09; *p* > 0.05), motivation (*t* = 0.09; *p* > 0.05), science scores (*t* = 0.01; *p* > 0.05), or mathematics scores (*t* = 0.19; *p* > 0.05).

Table 2 presents descriptive statistics, including means and standard deviations (SD), for SEL competencies, motivation, and science and mathematics scores as reported in the school records. The performance of all variables was acceptable, with no ceiling or floor effect. Skewness and kurtosis analyses confirmed normal distribution for all measures.

### 3.1. Effect of Integrating a SEL Program on Cognitive and Social–Emotional Measures

To address our main research question regarding the effect of integrating a SEL programme on cognitive and social–emotional measures, we conducted four separate repeated measures ANOVAs. These analyses examined variations in participant performance across SEL competencies, motivation, science scores, and mathematics scores, with time (pre- and post-intervention) as a ‘within-subject’ factor, and group (intervention vs. control) as a ‘between-subjects’ factor. The interaction between these factors was also examined to determine differential effects of the intervention between groups. Partial eta-squared (*η*^2^) values were interpreted against Cohen’s (1988) thresholds: *η*^2^ ≈ 0.01, 0.06, and 0.14 for small, medium, and large effects. Before each ANOVA, normality and sphericity were checked. Because the within-subjects factor (time) had only two levels, sphericity did not apply and no corrections (Greenhouse–Geisser or Huynh–Feldt) were needed. Table 3 presents the results of these analyses.

**Figure 1:**
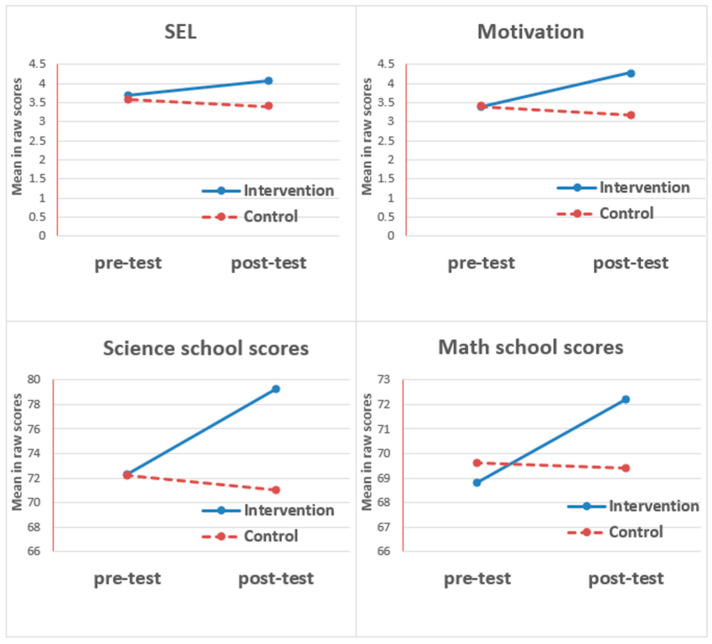
Performance in raw scores for dependent variables by group and time.

Because the study used a pre–post design with an intervention and a control group, intervention efficacy is best evaluated through the time × group interaction, rather than through the main effect of time. The main effect of the intervention on SEL competencies was not significant, indicating that participant performance, regardless of group, did not significantly change from pre-test (*M* = 3.65) to post-test (*M* = 3.73). However, a significant main effect of group emerged, with the intervention group (*M* = 3.89) outperforming the control one (*M* = 3.50), regardless of time. The significant interaction between the two factors may be attributed to a significant increase in the intervention group’s scores from pre-test to post-test (*t* = 4.19; *p* < 0.001), while the control group showed no significant changes (see Figure 1).

The same logic applies to the science scores: the main effect of time was not significant, with similar overall performance at post-test (*M* = 74.9) and pre-test (*M* = 72.3). The main effect for group, regardless of time, failed to reach statistical significance, showing only a slight difference between the intervention (*M* = 75.8) and control (*M* = 71.6) groups. However, a significant interaction effect was found, which can be attributed to significant improvement between pre-test and post-test for the intervention program group (*t* = 3.25; *p* < 0.01), but not the control group. Regarding mathematics scores, no significant main effects of time or group were observed. Performance did not differ significantly between pre-test (*M* = 69.2) and post-test (*M* = 70.7), regardless of group, or between intervention group (*M* = 70.5) and control group (*M* = 69.5), regardless of time. Unlike the science scores, the interaction between the two factors was not significant for mathematics scores, suggesting similar patterns of change across both groups (see Figure 1). Using Cohen’s (1988) benchmarks, the time × group interaction was large for motivation towards science (*η*^2^ = 0.48) and approached the large-effect threshold for SEL competencies (*η*^2^ = 0.11). It fell in the medium range for science achievement (*η*^2^ = 0.09) and in the small range for mathematics (*η*^2^ = 0.03), in keeping with the domain-specific design of the intervention.

### 3.2. Impacts of the SEL on Typically Developing Children Versus Those with RD

To address the second part of our research question concerning differential impacts of the intervention program on typically developing children versus those with RD, we again conducted separate repeated measures ANOVAs for each variable. Results are presented in Table 4. A significant main effect was observed only for time, with higher performance in the post-test (*M* = 3.70) than in the pre-test (*M* = 3.40). No significant differences between pre-test and post-test were found for SEL competencies, science scores, or mathematics scores. A significant main effect of group (*p* < 0.001) was obtained across all measures, with the typically developing group consistently outperforming the RD group. None of the time × group interactions reached significance across the outcome measures in these subgroup analyses. The reader subgroups were fairly small (*n* = 51 typically developing, *n* = 50 with RD), and the present analyses did not include formal equivalence or Bayesian tests. The non-significant interactions should therefore be read as inconclusive, not as evidence that the two subgroups responded equivalently to the intervention.

## 4. Discussion

In the current study, the impact of integrating a SEL intervention programme into science instruction for Arabic-speaking sixth-grade students was examined. Significant time × group interactions were found for SEL competencies, motivation towards science, and science achievement, though not for mathematics (see Table 3 and Figure 1). Post hoc comparisons showed that these interactions reflect pre- to post-test gains in the intervention group only; the control group did not improve to a comparable extent. The subgroup analyses did not detect a differential response to the intervention between typically developing children and those with RD. However, two features of the data constrain what this null finding can tell us: the reader subgroups were small, and no formal equivalence testing was carried out (see Section 4.1). These findings provide strong evidence that SEL integration in science education can foster both cognitive and social–emotional growth, with benefits for learners facing linguistic and cognitive challenges. The large time × group interaction for motivation and the near-large effect for SEL competencies are on par with the effect sizes commonly reported in meta-analyses of universal school-based SEL interventions (Cipriano et al., 2023). The medium effect on science achievement sits within the more moderate range that academic-transfer effects in this literature tend to show.

The observed enhancement in SEL competencies among students in the intervention group is consistent with a substantial body of prior research demonstrating that structured, curriculum-integrated SEL programs promote growth in self-awareness, self-regulation, social awareness, and interpersonal skills (Cipriano et al., 2023; Durlak et al., 2022). In line with the CASEL framework (Collaborative for Academic, Social, and Emotional Learning, 2023), the present findings suggest that embedding SEL practices within subject-matter instruction, rather than treating them as an isolated component, creates meaningful opportunities for students to actively practise emotional regulation, perspective taking, and collaborative problem-solving in authentic learning contexts. Similar gains have been reported in science-based SEL interventions that incorporate reflective dialogue and cooperative learning, which have been shown to strengthen classroom climate and students’ sense of engagement (Gericke et al., 2022; Hofstein & Mamlok-Naaman, 2007).

From an interpretive perspective, these findings indicate that the improvement in SEL competencies is not merely an ancillary outcome of the intervention, but rather a mechanism through which learning environments become more supportive of sustained engagement and self-regulated learning. As discussed in the literature review, social–emotional competencies are closely linked to motivation, persistence, and the ability to cope with cognitively demanding tasks (Duckworth & Seligman, 2005; Ryan & Deci, 2009).

A conceptual framework clarifies how the SEL-integrated intervention could plausibly influence cognitive and academic outcomes, and why its effects appeared in science but not in mathematics. Social–emotional competencies do not replace cognitive resources such as working memory and executive function; rather, they act as regulatory processes that shape how efficiently those resources are used during learning (Baddeley, 2003; Duckworth & Seligman, 2005; Ryan & Deci, 2009). Self-awareness and self-management ease the cognitive load imposed by unregulated emotional reactions to challenging material, freeing working memory for encoding, integrating, and manipulating new content. Relationship skills and social awareness, in turn, support the executive functions that cooperative inquiry draws on: planning, attention control, perspective-taking, and flexible shifting between individual and joint goals. These regulatory and collaborative mechanisms map onto the cognitive and epistemic demands of science learning, which calls for sustained engagement with text-based and abstract content, the coordination of multiple sources, and extended discussion and reflection (Fang, 2006; Hofstein & Mamlok-Naaman, 2007). Mathematics at this age rests more heavily on symbolic and procedural operations whose efficiency turns on domain-specific numerical skills; such operations are less prone to affective interference and gain less from collaborative, discussion-based scaffolding. Transfer of the SEL intervention effects to science was therefore theoretically expected. Non-transfer to mathematics sits comfortably with the more limited overlap between the regulatory and relational resources targeted by SEL and the symbolic and procedural processes central to sixth-grade mathematics.

Within this framework, SEL is best understood not as a statistical mediator of academic achievement but as an instructional design principle that sets a contextual condition for learning: by embedding opportunities for self-awareness, self-management, and relational engagement in the scientific content, SEL-integrated instruction changes the affective and collaborative conditions under which cognitive resources are used, shaping the efficiency of learning, rather than directly producing it. However, this interpretation calls for caution. The intervention was embedded only in science instruction, with no parallel change in mathematics teaching; the absence of gains in mathematics is therefore at least partly a direct consequence of the design, not evidence that SEL has domain-specific limits. The conceptual contrast between science and mathematics is best read as a plausible account of why transfer was unlikely in the present study, not as a test of whether SEL can support mathematics learning when explicitly integrated into mathematics instruction. That latter question awaits a dedicated, discipline-matched intervention.

The significant increase in motivation towards science in the intervention group supports motivational theories which emphasise that social–emotional support and meaningful engagement in content boost intrinsic motivation and persistence in challenging learning areas (Ryan & Deci, 2009; Wigfield et al., 2016). The integration of SEL into science lessons addresses social–emotional barriers that often hamper learning, particularly for students struggling with unfamiliar and complex scientific vocabulary and concepts (Basheer et al., 2025a; Perfetti & Stafura, 2014; Asadi & Asli-Badarneh, 2023).

In the context of science learning, particularly in linguistically complex settings such as Arabic-speaking classrooms, SEL may function as a regulatory scaffold that enables students to manage frustration, navigate challenging texts, and participate more actively in collaborative inquiry. The present results thus extend previous findings by demonstrating that SEL integration within science instruction can simultaneously enhance social–emotional development and create conditions conducive to deeper academic engagement, for both typically developing and RD students. This interpretation is in line with neuroscientific work showing that language and social cognition involve partially overlapping neural networks and have co-evolved as interconnected capacities (Oesch, 2024). From this perspective, the gains observed in the intervention group may reflect shared socio-linguistic processes being mobilised by instructional contexts that explicitly engage both domains.

That said, academic gains in science achievement, but not in mathematics, highlight the domain-specific nature of the intervention. The SEL-based activities implemented in the present study appear to have facilitated deeper engagement with scientific content by fostering students’ emotional involvement, sense of competence, and active participation in the learning process. Rather than merely supporting content acquisition, the integration of SEL into science instruction likely enhanced students’ commitment to learning by creating an environment in which curiosity, collaboration, and reflective thinking were explicitly encouraged. Prior research suggests that such emotionally supportive and socially meaningful learning contexts promote sustained attention, willingness to engage with challenges, and deeper conceptual processing, processes that are central to successful science learning (Ryan & Deci, 2009; Wigfield et al., 2016).

In contrast to mathematics learning, which often relies on abstract and symbolic representations and was not directly addressed by the intervention, science instruction in this study offered concrete phenomena, collaborative inquiry, and opportunities for discussion and reflection. These features align closely with SEL principles and may explain why gains were observed specifically in science achievement. As argued in the literature, science learning places distinct cognitive and epistemic demands on students, requiring them not only to comprehend complex texts but also to negotiate meaning, justify explanations, and integrate new knowledge with prior experience (Fang, 2006; Hofstein & Mamlok-Naaman, 2007). Embedding SEL practices within this process may therefore strengthen students’ sense of ownership and relevance, leading to deeper engagement and improved retention of scientific concepts.

Although typically developing children outperformed their peers with RD across all measures, the group × time interactions were not significant in the subgroup analyses, a pattern broadly compatible with, though not formal evidence for, the possibility that SEL-infused science instruction can serve as an equitable educational approach, supporting diverse learners by addressing both cognitive and social–emotional dimensions of learning (Basheer et al., 2025a; Stevens et al., 2021).

The findings reaffirm the integrative perspective of academic achievement being influenced by an interplay of cognitive capacities and motivational and social–emotional processes (Cipriano et al., 2023; Duckworth & Seligman, 2005). The improvement in scientific achievement through SEL integration supports the Simple View of Reading framework (Gough & Tunmer, 1986) while extending it to additional social–emotional variables, as reflected by the role of SEL competencies in enabling learners to access and comprehend challenging scientific texts. Moreover, the intervention’s focus on self-regulation resonates with Vygotsky’s (1978) social constructivist theory, which posits that cognitive development is mediated through social interaction and internalisation of language and regulatory tools.

These results underscore the importance of embedding SEL in science curricula, particularly in linguistically diverse contexts such as Arabic-speaking classrooms, where diglossia may further influence social–emotional processing (Asli-Badarneh, 2025; Asadi & Asli-Badarneh, 2023). Educators should consider employing experiential, hands-on learning activities alongside explicit SEL instruction to create enriched learning environments. The findings advocate for professional development initiatives that equip teachers to integrate SEL frameworks within the subject that they are teaching, fostering both academic (cognitive) and social–emotional (non-cognitive) competencies. Furthermore, differentiated instruction that accommodates learners’ varying abilities, including those with RD, can enhance inclusivity and equity.

### 4.1. Limitations

The study has several limitations. First, the design was confined to a single grade level and a relatively short intervention, and the data have a nested structure—four intact classes across two schools—that would normally call for MLM; with so few clusters, well below the minimum recommended for stable estimation of level 2 parameters (Maas & Hox, 2005), MLM was not suitable, and the analyses were run at the individual level. Second, RD was operationalised via a median split on a curriculum-based composite score of reading accuracy, fluency, and comprehension, consistent with an RTI-aligned approach (Fuchs et al., 2004; Vellutino et al., 1996). This is not a clinical diagnosis of dyslexia or a specific learning disability, and findings on differential responses should be read as descriptive of performance-based subgroups, not of clinically defined populations. Two further statistical costs follow: treating reading ability as two categorical subgroups rather than as a continuous, potentially multi-dimensional construct reduces statistical information; and with 51 and 50 children per subgroup, the analyses were underpowered to detect small-to-moderate time × group interactions and did not include equivalence or Bayesian tests. Non-significant subgroup interactions should therefore be read as inconclusive, not as evidence that typically developing children and those with RD responded equivalently. Third, the control condition was a business-as-usual comparison matched on time on task, content, and instructor profile, rather than an active SEL-free alternative intervention; this design cannot fully isolate the SEL components from novelty or heightened attention in the intervention classrooms. Implementation fidelity was supported by a single teacher per intervention classroom, joint planning with the school’s educational counsellor, and ongoing contact with the research team, but the study did not include a formal fidelity-of-implementation check by independent external observers. Fourth, outcomes were based on teacher-constructed, curriculum-based assessments drawn from school records rather than on standardised, externally administered tests. This preserves ecological validity and reflects the scores that routinely inform educational decisions, but it introduces between-teacher variability and some uncontrolled subjectivity in scoring. A related measurement gap is that only mathematics achievement, and not motivation towards mathematics, was assessed. Finally, because the intervention was delivered by a single teacher in each of the two schools, teacher effects and school-level characteristics are inherently confounded with the intervention, which limits the extent to which observed differences can be attributed to the SEL components rather than to specific teacher or site characteristics. The absence of mathematics gains should therefore be read as a constraint of the intervention design, which embedded SEL components exclusively within science instruction, rather than as evidence of domain-specific limits of SEL transfer.

### 4.2. Future Research

Several research directions follow from these limitations. First, the design should be replicated across grade levels and with longer durations, including longitudinal follow-up, to assess the sustainability and developmental specificity of the observed gains. Second, replication with standardised, multi-dimensional diagnostic tests validated for Arabic speakers and with formally diagnosed samples would establish whether the patterns observed here generalise to children with clinically confirmed reading disorders; within such designs, subgroup comparisons should rely on larger samples and pre-registered equivalence or Bayes-factor tests, and reading ability would be better treated as a continuous, multi-dimensional variable entered directly into regression or mixed-effects models, which would also increase statistical power. Third, studies with a larger number of classes and schools should apply multi-level or mixed-effects models and incorporate structured fidelity measures, such as coded observations of a subset of sessions or independent ratings of lesson implementation. Fourth, an active attention-control condition delivering a content-matched program without the SEL elements would more stringently isolate the contribution of SEL integration itself. Fifth, subsequent studies should include a motivation measure for mathematics alongside achievement, to examine possible affective effects across domains. Finally, qualitative measures would add depth with respect to students’ subjective experiences and the mechanisms through which SEL-integrated instruction operates in different academic domains.

## 5. Conclusions

The present study provides experimental evidence that a curriculum-integrated SEL intervention, delivered by regular classroom teachers within sixth-grade science instruction, can produce meaningful gains for Arabic-speaking children from linguistically and socio-economically marginalised communities. Over a 10-week program built around the five CASEL competencies, the intervention produced large gains in motivation towards science and near-large gains in SEL competencies, a medium-sized gain in science achievement, and no corresponding gains in mathematics. This pattern fits a framework in which SEL operates as a regulatory resource well suited to the text-based, collaborative, and discussion-intensive work of science, and less suited to the symbolic and procedural operations that dominate mathematics at this age. The absence of any SEL effects’ transfer to mathematics is also partly a consequence of the design itself: the intervention was embedded only in science. Whether typically developing readers and those with RD responded differently to the intervention remains an open question and needs confirmation with larger, formally diagnosed samples and pre-registered equivalence tests. On balance, the findings support SEL-integrated science instruction as a culturally responsive pedagogical approach and warrant its consideration in teacher preparation and curriculum-design initiatives for elementary science education in multi-lingual settings.

## Figures and Tables

**Table 1:** Representative Examples of SEL Activities Embedded in the Sixth-Grade Science Curriculum, Mapped to the Five CASEL Competencies.

CASEL Competency	Representative Scientific Topic	Example SEL Activity
Self-awareness	Communication systems in the body (sensory organs, nervous system)	Open-ended question: “What would you feel if you could no longer see or hear?”; children articulate personal reactions and link them to the scientific content on sensory processing
Self-management	Electrical energy and magnetism (circuit-building tasks)	Group experiments that sometimes yield unexpected results; children name and regulate frustration, decide how to retry, and reflect on persistence
Social awareness	Sound energy and animal communication	Perspective-taking: “How might animals feel when they cannot communicate?”; children articulate empathy and extend it to classmates with different communication styles
Relationship skills	Ecological systems and interactions between organisms	Small-group investigations of ecological relationships requiring negotiation of roles and joint decision making; peer discussion of cooperation versus competition
Responsible decision-making	Human impact on the environment and sustainable development	Structured debate on pro-environmental choices; group commitment to a small everyday action; reflective closing on personal and collective responsibility

**Table 2:** Descriptive statistics for all variables and school scores (*n* = 101).

Variables	Mean (*SD*)	Min	Max	Skewness	Kurtosis
SEL	3.63 (0.58)	2.10	4.58	−0.50	−0.40
Motivation	3.40 (0.81)	1.0	5.60	−0.14	0.36
Science school scores	72.3 (21.5)	20	100	−0.58	−0.57
Maths school scores	69.2 (20.9)	40	100	−0.01	−1.40

Abbreviation: SEL = social–emotional learning skills.

**Table 3:** Descriptive statistics of raw scores (mean and SD) with ANOVA * by group and time.

	Groups						
	Intervention ^a^		Control ^b^		ANOVA		
	pre	post	pre	post	Time	Group	Time × Group
	*M*	*M*	*M*	*M*	*F* (1, 100)	*F* (1, 100)	*F* (1, 99)
	(*SD*)	(*SD*)	(*SD*)	(*SD*)	(*η*^2^)	(*η*^2^)	(*η*^2^)
SEL	3.70	4.08	3.59	3.40	1.36 ^ns^	15.2 ***	11.8 ***
	(0.55)	(0.46)	(0.57)	(0.88)	(0.01)	(0.14)	(0.11)
Motivation	3.39	4.28	3.40	3.16	28.6 ***	16.6 ***	88.9 ***
	(0.83)	(0.61)	(0.80)	(0.70)	(0.23)	(0.15)	(0.48)
Science *	72.3	79.3	72.2	71.0	3.9 ^ns^	1.1 ^ns^	9.4 **
	(23.5)	(21.9)	(19.5)	(18.8)	(0.04)	(0.01)	(0.09)
Maths *	68.8	72.2	69.6	69.4	2.2 ^ns^	0.6 ^ns^	2.8 ^ns^
	(20.4)	(20.3)	(21.6)	(21.0)	(0.02)	(0.01)	(0.03)

Abbreviation: SEL = social–emotional learning skills. * Science and maths represent school scores. ^a^ *n* = 50; ^b^ *n* = 51. ns = nonsignificant. ** *p* < 0.01 *** *p* < 0.001.

**Table 4:** Descriptive statistics of raw scores (mean and SD) with ANOVA * by time and group.

	Groups						
	Typical ^a^		RD ^b^		ANOVA		
	pre	post	pre	post	Time	Group	Time × Group
	*M*	*M*	*M*	*M*	*F* (1, 100)	*F* (1, 100)	*F* (1, 99)
	(*SD*)	(*SD*)	(*SD*)	(*SD*)	(*η*^2^)	(*η*^2^)	(*η*^2^)
SEL	3.86	3.93	3.41	3.52	0.95 ^ns^	19.2 ***	0.03 ^ns^
	(0.54)	(0.66)	(0.48)	(0.85)	(0.01)	(0.17)	(0.00)
Motivation	3.70	3.98	3.08	3.41	13.5 ***	19.5 ***	0.12 ^ns^
	(0.77)	(0.81)	(0.74)	(0.84)	(0.12)	(0.17)	(0.00)
Science *	82.6	84.3	61.3	65.0	3.4 ^ns^	35.1 ***	0.45 ^ns^
	(17.3)	(16.2)	(20.1)	(20.5)	(0.03)	(0.27)	(0.01)
Maths *	80.4	81.4	57.4	59.4	2.1 ^ns^	44.9 ***	0.20 ^ns^
	(18.4)	(17.8)	(16.7)	(17.2)	(0.02)	(0.32)	(0.00)

Abbreviation: SEL = social–emotional learning skills. * Science and maths represent school scores. ^a^ *n* = 51; ^b^ *n* = 50. ns = nonsignificant. *** *p* < 0.001.

## Data Availability

The datasets generated and analysed during the current study are available from the corresponding author upon reasonable request. The data are not publicly accessible because they contain information related to school-aged participants and are subject to the ethical approval and parental consent conditions under which the study was conducted (Arab Academic College of Education in Israel; Approval Code: AACE-SE-91224.3).
